# In Situ Sonoactivation of Polycrystalline Ni for the Hydrogen Evolution Reaction in Alkaline Media

**DOI:** 10.1021/acsaem.2c02443

**Published:** 2023-04-21

**Authors:** Faranak Foroughi, Marina Tintor, Alaa Y. Faid, Svein Sunde, Gregory Jerkiewicz, Christophe Coutanceau, Bruno G. Pollet

**Affiliations:** † Hydrogen Energy and Sonochemistry Research Group, Department of Energy and Process Engineering, Faculty of Engineering, Norwegian University of Science and Technology (NTNU), Trondheim NO-7491, Norway; ‡ Department of Chemistry, Queen’s University, 90 Bader Lane, Kingston, Ontario K7L 3N6, Canada; § Electrochemistry Research Group, Department of Materials Science and Engineering, Faculty of Natural Sciences, Norwegian University of Science and Technology (NTNU), Trondheim NO-7491, Norway; ∥ Catalysis and Non-Conventional Medium group, IC2MP, UMR CNRS 7285, Université de Poitiers, 4 Rue Michel Brunet, 86073 Cedex 9 Poitiers, France; ⊥ French Research Network on Hydrogen (FRH2), Research Federation n°2044 CNRS, BP 32229, 44322 Nantes CEDEX 3, France; # Green Hydrogen Lab, Institute for Hydrogen Research, Université du Québec à Trois-Rivières, 3351 Boulevard des Forges, Trois-Rivières, Québec G9A 5H7, Canada

**Keywords:** polycrystalline nickel, hydrogen evolution reaction, alkaline water electrolysis, ultrasound, sonoelectrochemistry, enhancement of electrocatalytic activity

## Abstract

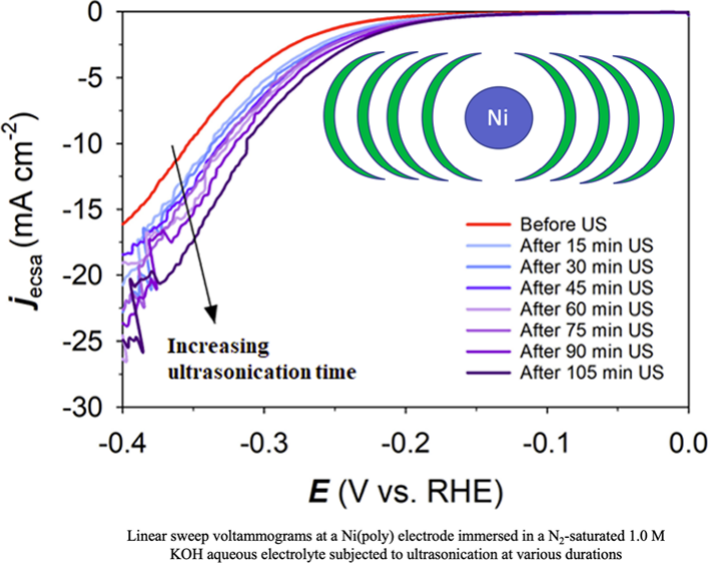

In this investigation, we report on the development of a method for activating polycrystalline metallic nickel (Ni(poly)) surfaces toward the hydrogen evolution reaction (HER) in N_2_-saturated 1.0 M KOH aqueous electrolyte through continuous and pulsed ultrasonication (24 kHz, 44 ± 1.40 W, 60% acoustic amplitude, ultrasonic horn). It is found that ultrasonically activated Ni shows an improved HER activity with a much lower overpotential of −275 mV vs RHE at −10.0 mA cm^–2^ when compared to nonultrasonically activated Ni. It was observed that the ultrasonic pretreatment is a time-dependent process that gradually changes the oxidation state of Ni and longer ultrasonication times result in higher HER activity as compared to untreated Ni. This study highlights a straightforward strategy for activating nickel-based materials by ultrasonic treatment for the electrochemical water splitting reaction.

## Introduction

Electrochemical water splitting (water electrolysis) technologies will play an important role in meeting the stringent climate change targets by producing molecular hydrogen (H_2_(g)) as a fuel and energy carrier, and these technologies will accelerate the transition toward a renewable and carbon-free energy society.^1^ Only a mere 1% of the hydrogen produced globally is produced through water electrolysis, while 96% is still generated through steam methane reforming (SMR) of carbonaceous sources.^1^ Water electrolysers coupled with intermittent renewable energy systems are well suited to provide the foundation of a sustainable hydrogen production network. Alkaline water electrolysis (AWE) is a well-established and mature technology for clean hydrogen generation offering several advantages, including (i) low initial capital expenditure (CAPEX) and operational expenditure (OPEX), (ii) a proven and scalable technology, (iii) the establishment of industrially large capacity units, and (iv) no requirement for extensive water purification procedures.^1^ Potassium hydroxide (KOH, 30–40%) is preferably used in AWE over sodium hydroxide (NaOH) due to its higher conductivity.^1,2^ The hydrogen evolution reaction (HER) in AWE occurs at the cathode of the electrolyser according to eq 1:
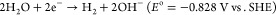
1

The HER in alkaline electrolytes is a complicated multistep electrochemical reaction occurring on the electrode surface and is known to proceed via a combination of three fundamental steps, namely, the Volmer, the Heyrovsky, and the Tafel steps. The HER pathway in alkaline media proceeds either through the Volmer–Heyrovsky or the Volmer–Tafel pathway as shown in Table 1, with the Volmer step being common to the two mechanistic pathways. The HER mechanisms can be inferred from the Tafel slope, which is determined from the linear portions of Tafel plots fitted to the Tafel equation.^3,4^

**Table 1:** HER Mechanisms in Alkaline Media^4,5^

mechanism	rate-determining step	Tafel slope mV dec^–1^
Volmer–Heyrovsky	H_2_O + e^–^ → H_ads_ + OH^–^	120
	Volmer	
Volmer–Heyrovsky	H_ads_ + H_2_O + e^–^ → H_2_ + OH^–^	40
	Heyrovsky	
Volmer–Tafel	2H_2_O + 2e^–^ → 2H_ads_ + 2OH^–^	60
	Volmer	
Volmer–Tafel	2H_ads_ → H_2_	30
	Tafel	

Nickel (Ni) and Ni-based materials (e.g., Ni-Raney) are common choices as electrode materials in AWE due to their low cost, good catalytic activity, and availability.^4,6,7^ However, Ni materials require further improvements in catalytic performance to meet the technical challenges of AWEs, such as lower catalytic performance and higher system resistance compared to proton exchange membrane water electrolysis.^1^ Several strategies have been adopted to Ni-based catalysts with significant benefits, e.g., downsizing to the atomic scale,^8^ alloying with other elements,^9,10^ generating heterojunctions,^11,12^ or spontaneous deposition of Ru and Ir.^13^ Among these methodologies, the formation of Ni heterostructures, specifically Ni/NiO or Ni/Ni(OH)_2_, is of great interest as the synergy between Ni and NiO has shown to enhance HER performance.^14−18^ Oshchepkov et al. found that Ni/NiOx with a maximum at 30% NiOx yields the optimum HER activity.^17^ A layer of NiO/Ni(OH)_2_ provides oxophilic sites that facilitate and enhance the rate of the Volmer step of the HER, which results in the formation of H_ad_ and OH^–^.^19,20^ In fact, Markovic and co-workers showed that adding Ni(OH)_2_ onto metal surfaces that include Ni yields a 3- to 5-fold enhancement in the HER rate.^19^

Power ultrasound is a well-defined sound wave in the 20 kHz–2 MHz ultrasonic frequency range. It is well known that the propagation of an ultrasonic wave into a liquid leads to acoustic cavitation.^24^ The use of ultrasound in electrochemistry, also known as *sonoelectrochemistry*, yields: (a) an area of extreme mixing within the area of the ultrasonic transducer, (b) electrode and electrolyte degassing, (c) electrode erosion and cleaning, thus activation, and (d) an increase in the electrolyte bulk temperature, (e) acoustic cavitation (creation and implosion of cavitation bubbles on the electrode surface), (f) the production of highly reactive radicals (e.g., H^·^ and OH^·^) and hydrogen peroxide (H_2_O_2_), also known as water sonolysis (see the Supporting Information for the complete water sonolysis chemical reactions), and (g) sono(electro)chemiluminescence.^21−23^ It is also known that ultrasonication greatly improves the electrocatalytic properties of metallic surfaces due to cavitation erosion and cleaning induced by high-velocity jets of liquid generated by the implosion of cavitation bubbles on/near the surfaces.^24−29^

We initially investigated the effects of ultrasonication on polycrystalline platinum, Pt(poly), in acidic electrolytes and on Raney-Ni in alkaline electrolytes and observed that the HER was greatly enhanced.^30,31^ In these studies, continuous ultrasonication was employed during the electrochemical HER and OER experiments.

In this study, the surface state and the electrocatalytic activity of the sonoactivated Ni(poly) electrode toward the HER are evaluated by cyclic voltammetry (CV) and linear sweep voltammetry (LSV) measurements. The influence of the duration of the ultrasonic (US) treatment on the surface morphology of Ni(poly) is analyzed by scanning electron microscopy (SEM). To clarify, in this investigation continuous ultrasonication was not applied during the electrochemical HER and OER experiments.

The activation procedure that we are proposing is simple and involves a one-step ultrasonication (24 kHz, 60% amplitude, either continuous or pulsed mode) in 1.0 M aqueous KOH solution, and to the best of our knowledge, there is no report in the literature on the in situ activation of Ni(poly) electrodes toward the HER using power ultrasound, hence the originality of this contribution and its possible importance to the AWE technology. It should be emphasized that sonoactivation of Ni(poly) electrodes that we are herein proposing is not competing with other methodologies to activate the Ni surfaces such as “electro-oxidation” but can be seen as complementary. One of the many advantages of ultrasonication is the enhanced electrode surface cleaning.^1^

## Results and Discussion

### Scanning Electron Microscopy Characterization of Polycrystalline Ni before and after Ultrasonication

**Figure 1:**
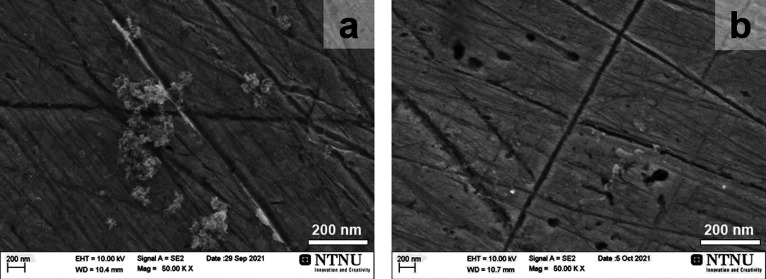
SEM images of (a) Ni(poly) electrode before ultrasonication and (b) after 105 min ultrasonication.

The SEM image of the Ni(poly) surface that was polished with gradually smaller alumina particles (from 5 μm down to 0.05 μm in diameter) is shown in Figure 1a. The image displays scratches due to mechanical polishing and a few residual alumina particles. After ultrasonication in 1.0 M aqueous KOH solution for 105 min, a few irregularly shaped pits are visible on the ultrasonicated electrode (Figure 1b); because of their small size and low density, they make a relatively small contribution to the total electrochemical surface area (*A*_ecsa_). Thus, surface roughening due to ultrasonication of the Ni(poly) electrode was found to be minor under the conditions reported in this contribution. It is an interesting and intriguing finding as it is well known in the area of sonoelectrochemistry that power ultrasound together with induced cavitation leads to surface damage and deformations.^32−34^ One possible explanation for our observation may be due to the properties of Ni, and in particular, its hardness.

In the 1990s, Marken et al. showed that ultrasound affects the surfaces of gold (Au) and glassy carbon (GC) electrodes.^34^ They observed that after a period of ultrasonic treatment, severe surface damage was observed for both Au and GC electrodes with a roughening in the 10 μm scale as well as in a smaller scale with ca. 0.1 μm sized pits. They found that this type of severe damage occurred only after high-intensity power ultrasound continuous exposure (20 kHz, 63 W cm^–2^) and at a close distance between the electrode and the ultrasonic horn (<35 mm) immersed in the electrolyte (known in sonoelectrochemistry as the “face-on” geometry). Using atomic force microscopy (AFM), CV, and electrochemical impedance spectroscopy (EIS), they also found that the roughening and the capacitance of the Au surface as compared to the GC surface also appeared to be considerably lower under these conditions.

Contrarily to the work of Marken et al., Madigan et al. showed that the electrode surface roughness remained almost unchanged for some electrode materials after ultrasonication.^35^ They studied a series of nonmetallic and metallic electrodes under 20 kHz ultrasonic exposure in aqueous electrolytes. They found that GC and Ebonex were severely pitted after only a few minutes of ultrasonication in aqueous media, while Pt, Pd, Au, and W remain largely undamaged after 120 s, as observed by SEM. They concluded that surface damage is more closely related to the hardness of the material.

To demonstrate the extent of ultrasonication in our sonoelectrochemical cell, a simple experiment, using pieces of aluminum (Al) foil, was conducted to determine the degree of acoustic cavitation on the Al surface. Since Al is a malleable and ductile metal, when immersed in water in the presence of power ultrasound, pinholes usually appear after a few minutes and even seconds, indicating the implosion of cavitation bubbles on its surface. The experimental conditions are fully described in the Supporting Information section (Figures S2 and S3), and the results show that, under the conditions reported in this contribution and using the experimental setup, ultrasound did not greatly affect the Al surface, i.e., no pinholes were observed. Sonochemical dosimetry experiments (see the Supporting Information, Water Sonolysis) were also carried out, although the findings were not conclusive as an indicator for the production of hydrogen peroxide (H_2_O_2_) and radical species formation such as OH radicals. This is probably due to the limitations of the dosimetry methodology (detection limits). It could also imply that no “observable” cavitation occurred in our “nonoptimized” sonoelectrochemical setup, in which the ultrasonic horn is separated from the inner electrochemical cell at a distance *d* of 30 mm (the ultrasonic horn is facing the base of the inner electrochemical cell made of a thin glass) and thus it is not immersed directly in the electrolyte and placed very close to the electrode, contrarily to the other studies^34,35^ (see Supporting Information, Figure S1).

### Analysis of the Effect of the Ultrasonic Treatment Duration on the Electrochemical Surface Area (*A*_ecsa_)

**Figure 2:**
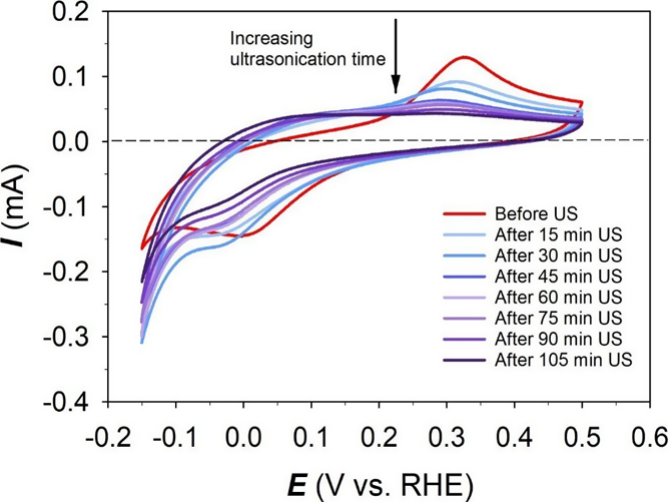
Cyclic voltammograms of the Ni(poly) electrodes in N_2_-saturated 1.0 M aqueous KOH solution acquired at ν = 100 mV s^–1^ and *T* = 298 K before and after US at various ultrasonication times (*A*_ecsa_ = 0.35 cm^2^).

Figure 2 shows CV transients of the Ni(poly) electrode in the potential region of the formation and reduction of α-Ni(OH)_2_ (−0.15 ≤ *E*_app_ ≤ +0.50 V vs RHE) at a potential scan rate of ν = 100 mV s^–1^ and *T* = 298 K before and after 15, 30, 45, 60, 75, 90, and 105 min of ultrasonication. The CV profile prior to ultrasonication shows that the Ni(poly) electrode is metallic (for clarity and consistence of presentation, all CV profiles, polarization curves, referring to the untreated (nonsonicated) Ni(poly) electrode are shown as red). However, the gradual decrease of the CV features associated with the formation and reduction of α-Ni(OH)_2_ and their eventual disappearance indicate that the Ni(poly) electrode surface becomes oxidized (covered with a layer of β-Ni(OH)_2_).

This is an interesting finding which is in good agreement with those observed by Compton et al.^36^ They studied the effect of ultrasonication on surface electrochemical processes and the passivation of Ni electrodes in air-saturated 1.0 M aqueous KOH solution in which the Ni electrode was immersed and subjected to an anodic treatment at a potential scan rate of 100 mV s^–1^ in the absence (*silent* conditions) and presence of ultrasonication (20 kHz). Under *silent* conditions, the subsequent CVs showed first an increase in the anodic current as Ni oxidized and then a fast passivation of the Ni electrode surface at potentials more positive than the *Fladé* potential (the potential at which the electrode abruptly switches from a passive state to an active state). This finding was caused by a thin surface film of oxygen atoms chemisorbed to the Ni surface. Under ultrasonication, they also observed that the current magnitude was unaffected, confirming the surface-bound nature of the oxidative process. However, they observed that the *Fladé* potential was shifted anodically by ∼60 mV in the presence of ultrasound, and they mainly attributed this anodic potential shift to the Ni cleaning and surface erosion effects of ultrasound, in turn removing species attached to its surface.

To shed some light on the oxidation state and the extent of surface oxidation of Ni(poly) under the conditions reported here, it is important to quantify it. The overall electrochemical surface area (*A*_ecsa_) usually consists of two components, namely, the area of the metallic part (*A*_m_) and the area of the oxidized part (*A*_ox_). The value of *A*_ecsa_ is given by eq 2:
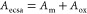
2

The surface area of metallic Ni(poly) (the CV profile prior to ultrasonication) is determined by calculating the charge (*Q*_α-Ni(OH)2_) associated with the formation of α-Ni(OH)_2_ and dividing it by the charge density (*q*_α-Ni(OH)2_) for the formation of a monolayer of α-Ni(OH)_2_, which is known to be 514 μC cm^–2^ (eq 3).^37^ However, the determination of *A*_ecsa_ through the α-Ni(OH)_2_ formation does not consider the Ni surface already covered with a layer of electrochemically formed oxide (e.g., β-Ni(OH)_2_ or β-NiOOH); thus, this approach requires that the entire Ni electrode surface to be in a metallic state prior to the formation of α-Ni(OH)_2_ or only the metallic portion of *A*_ecsa_ of the entire surface having both metallic patches and oxidized (passivated) ones is determined.^38^
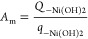
3

Because ultrasonication results in changes in the oxidation state of the Ni(poly) electrode (the extent of surface oxidation), *A*_ox_ can be calculated using eq 4, on the condition that the surface roughness remains unchanged after the ultrasonic treatment.
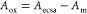
4

The validity of this important assumption was confirmed by SEM characterization of the Ni(poly) electrode before and after ultrasonication, which indicated that no noticeable surface roughening occurred during ultrasonication under our experimental conditions. Therefore, the surface roughness factor (RF) of the Ni(poly) electrode in all ultrasonication durations can be determined to be RF = 1.79 by using eq 5:
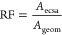
5where *A*_ecsa_ (cm^2^) is the electrochemical surface area and *A*_geom_ (cm^2^) is the geometric surface area (a two-dimensional projection of a roughened surface).

Prior to ultrasonication, the entire surface of the Ni(poly) electrode is metallic; thus, *A*_ecsa_ = *A*_m_ and *A*_ox_ = 0. However, after ultrasonication, the Ni(poly) electrode surface is partially oxidized and *A*_m_ is no longer equal to *A*_ecsa_; *A*_m_ is determined by eq 3, and *A*_ox_ is obtained by applying eq 4 to experimental data (the charge density associated with the oxide formation, which decreases as the duration of ultrasonication increases). The values of *A*_ecsa_, *A*_m_, and *A*_ox_ of the nonsonicated Ni(poly) electrode and after various ultrasonication times (15, 30, 45, 60, 75, 90, and 105 min) were calculated and are summarized in Table 2. Table 2 also shows the values of the fractions of the metallic and oxidized portions of the overall surface of the Ni(poly) electrode, *f*_m_ and *f*_ox_, where *f*_m_ = *A*_m_/*A*_ecsa_ and *f*_ox_ = *A*_ox_/*A*_ecsa_.

**Table 2:** Area of the Metallic Surface (*A*_m_), the Area of the Oxidized Surface (*A*_ox_), and the Electrochemical Surface Area (*A*_ecsa_) of the Ni(Poly) Electrode in 1.0 M KOH Aqueous Solutions before and after 15, 30, 45, 60, 75, 90, and 105 min US

	*A*_m_ (cm^2^)	*A*_ox_ (cm^2^)	*A*_ecsa_ (cm^2^)
before US	0.351 ± 0.007	0.000	0.350 ± 0.010
after 15 min	0.291 ± 0.001	0.059 ± 0.006	0.350 ± 0.010
after 30 min	0.271 ± 0.001	0.079 ± 0.011	0.350 ± 0.010
after 45 min	0.217 ± 0.017	0.133 ± 0.023	0.350 ± 0.010
after 60 min	0.216 ± 0.002	0.134 ± 0.036	0.350 ± 0.010
after 75 min	0.197 ± 0.003	0.153 ± 0.009	0.350 ± 0.010
after 90 min	0.180 ± 0.004	0.170 ± 0.002	0.350 ± 0.010
after 105 min	0.159 ± 0.005	0.191 ± 0.002	0.350 ± 0.010

The CV transient before ultrasonication illustrates typical features characteristic of metallic Ni,^17,37,38^ while the CV transients after ultrasonication show CV features characteristic of partially oxidized Ni.^17,38^ The anodic peak can be ascribed to the formation of α-Ni(OH)_2_ and the cathodic to its reduction to metallic Ni.^37^ The charge (size) of the anodic peak associated with the formation of α-Ni(OH)_2_ depends on the fraction of the Ni(poly) electrode that remains metallic (not passivated). Thus, as the fraction of the metallic surface decreases, so does the charge associated with the formation of α-Ni(OH)_2_ and, consequently, the value of *A*_m_.^16,37,38^ In general, ultrasonication is known to gradually increase the electrode surface area of metallic materials through roughening.^34^ Thus, one would expect the charge associated with the formation of α-Ni(OH)_2_ to be greater in the case of the Ni(poly) electrode being exposed to ultrasound, which is not the case here. In addition, our light microscopy and SEM analyses of the surface of the Ni(poly) electrode demonstrate that there is no noticeable surface roughening that could be attributed to the ultrasonic treatment (this is unique to the cell employed in this research). Consequently, the smaller size of this anodic CV peak as the duration of ultrasonication increases provides evidence that the Ni(poly) electrode undergoes gradual partial oxidation, the mechanism of which is discussed below.^38−41^

Our study now focuses on the Ni(poly) electrode surface state and the evolution of its morphology in relation to the duration of the ultrasonic treatment. This important knowledge is essential in the subsequent analysis of the influence of ultrasonication on the kinetics and mechanism of the HER.

### Study of the Effect of Ultrasonic Treatment Duration on the HER on a Polycrystalline Ni Electrode

**Figure 3:**
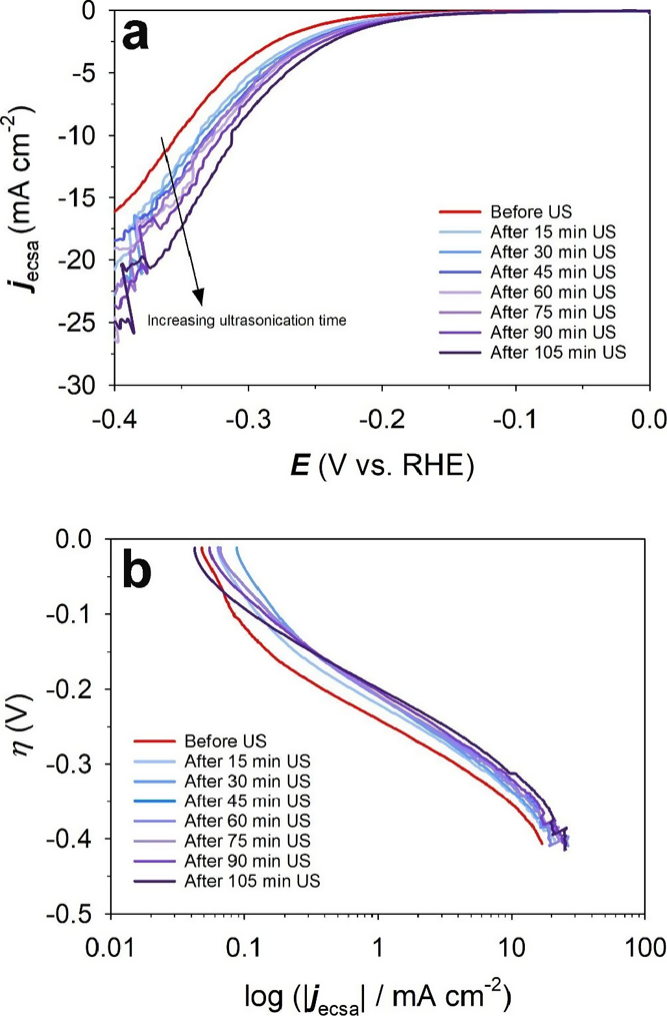
(a) Linear sweep voltammogram transients and (b) Tafel plots for the HER occurring at the Ni(poly) electrode in N_2_-saturated 1.0 M aqueous KOH solution prior to and after ultrasonication for various durations. The LSV transients were acquired at a potential scan rate of ν = 0.30 mV s^–1^ and at *T* = 298 K.

The electrocatalytic activity of the Ni(poly) electrode toward the HER prior to and after the ultrasonic treatment was evaluated by LSV. The LSV experiments were conducted at a very low potential scan rate to ensure that steady-state conditions are reached at each potential value.^42^Figure 3a shows LSV profiles of the Ni(poly) electrode in N_2_-saturated 1.0 M aqueous KOH solution in the 0.00 ≤ *E*_app_ ≤ −0.60 V vs RHE range and acquired at a potential scan rate of ν = 0.30 mV s^–1^ and at *T* = 298 K. It can be observed that before the ultrasonic treatment, the Ni(poly) electrode exhibits a low HER activity. However, the HER activity increases after ultrasonication, and the longer the ultrasonic treatment duration, the higher the electrocatalytic activity. The LSV transients were used to prepare Tafel plots (Figure 3b) to examine the kinetics and mechanism of the HER by determining the values of the Tafel slopes (*b*) and the exchange current density (*j*_o_). Because our results infer that the surface of the sonoactivated Ni(poly) electrode is partially covered by NiO/β-Ni(OH)_2_ while the rest of the surface is metallic, the HER may occur on both the metallic and oxidized sections of the Ni(poly) electrode but at different rates. Consequently, one may write:
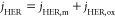
6where *j*_HER,m_ is the rate (the current density) of the HER at the metallic part and *j*_HER,ox_ is the rate (the current density) of the HER at the oxidized part of the Ni(poly) electrode. Consequently, the total rate of the HER (*j*_HER_) is the sum of these two contributions (eq 6). The evaluation of the area of the metallic surface (*A*_m_) and the area of the oxidized surface (*A*_ox_) allows us to determine *j*_HER,m_ and *j*_HER,ox_. The value of *j*_HER,m_ can be then calculated knowing the fraction of the metallic surface (*f*_m_ = *A*_m_/*A*_ecsa_) and using eq 7, and *j*_HER,ox_ can be calculated knowing the fraction of the oxidized surface (*f*_ox_ = *A*_ox_/*A*_ecsa_) and using eq 8:
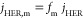
7
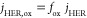
8

The values of *f*_m_, *f*_ox_, *j*_HER,m_, *j*_HER,ox,_ and *j*_HER_ at −300 mV vs RHE are reported in Table 3. This table also shows the Tafel slope (*b*), the rate of HER at *A*_m_ (*j*_HER,m_), the rate of HER at *A*_ox_ (*j*_HER,ox_), the total HER rate (*j*_HER_), at an overpotential of −10.0 mA cm^–2^ (*E*_–10.0mAcm_^–2^), and difference of overpotential (Δ*E* = *E*_US_ – *E*_before US_, with both potential values for −10.0 mA cm^–2^) for the HER on Ni(poly) electrode in 1.0 M aqueous KOH solution before and after 15, 30, 45, 60, 75, 90, and 105 min US.

**Table 3:** Tafel Slope (*b*), the Rate of HER at *A*_m_ (*j*_HER,m_), the Rate of HER at *A*_ox_ (*j*_HER,ox_), the Total HER Rate (*j*_HER_), at an Overpotential of −10.0 mA cm^–2^ (*E*_–10.0mAcm_^–2^), and Difference of Overpotential (Δ*E*) for the HER on Ni(Poly) Electrode in 1.0 M Aqueous KOH Solution before and after 15, 30, 45, 60, 75, 90, and 105 min US

	*E* @ −10 mA cm^–2^ (mV vs RHE)	Δ*E* @ −10 mA cm^–2^ (mV)	*f*_m_	*f*_ox_	*j*_HER,m at –300 mV_ (mA cm^–2^)	*j*_HER,ox at –300 mV_ (mA cm^–2^)	*j*_HER at –300 mV_ (mA cm^–2^)	*b* (mV dec^–1^)
before US	–353 ± 10		1.00	0.00	–3.95 ± 0.14	0.00	–3.95 ± 0.13	105 ± 17
after 15 min	–340 ± 20	13 ± 7	0.83 ± 0.01	0.17 ± 0.01	–3.28 ± 0.13	–1.97 ± 0.63	–5.25 ± 0.69	111 ± 8
after 30 min	–335 ± 16	18 ± 7	0.78 ± 0.02	0.22 ± 0.03	–3.08 ± 0.23	–2.80 ± 1.40	–5.88 ± 0.95	119 ± 11
after 45 min	–330 ± 18	23 ± 8	0.62 ± 0.05	0.38 ± 0.06	–2.45 ± 0.88	–4.02 ± 0.28	–6.47 ± 0.90	120 ± 9
after 60 min	–325 ± 16	28 ± 8	0.62 ± 0.01	0.38 ± 0.01	–2.45 ± 0.63	–4.44 ± 1.64	–6.89 ± 1.01	120 ± 4
after 75 min	–330 ± 22	23 ± 5	0.57 ± 0.02	0.43 ± 0.02	–2.25 ± 0.19	–4.39 ± 1.58	–6.64 ± 1.39	120 ± 10
after 90 min	–320 ± 25	33 ± 11	0.52 ± 0.01	0.48 ± 0.01	–2.05 ± 0.31	–5.31 ± 2.12	–7.36 ± 1.81	120 ± 12
after 105 min	–312 ± 23	41 ± 10	0.46 ± 0.01	0.54 ± 0.02	–1.82 ± 0.34	–6.59 ± 2.24	–8.41 ± 2.10	119 ± 7

The results show that the total rate of the HER expressed as the overall (total) current density (*j*_HER_) increases with increasing the ultrasonication duration, from −3.95 mA cm^–2^ before sonoactivation to −8.41 mA cm^–2^ after 105 min of ultrasonication. The rate of HER at the oxidized part of the Ni(poly) electrode surface (*j*_HER,ox_) is 0.00 mA cm^–2^ before ultrasonication since the entire surface of the Ni(poly) electrode is metallic, while after ultrasonication, the surface becomes gradually oxidized leading to an increasing rate of the HER at the oxidized part of the Ni(poly) electrode (*j*_HER,ox_). The value of *j*_HER,ox_ increased from 0.00 mA cm^–2^ before ultrasonication to reach −6.59 mA cm^–2^ after 105 min of ultrasonication, while the rate of the HER at the metallic part of the Ni(poly) electrode (*j*_HER,m_) decreased from −3.95 mA cm^–2^ before ultrasonication to −1.82 mA cm^–2^ after 105 min of ultrasonication. A synergistic interaction between the metallic Ni(poly) electrode and its surface oxide/hydroxide has been suggested as the main cause for the enhanced HER performance. For example, Strmcnik et al.^43^ showed that the metal/oxide interface plays an essential role in the water dissociation where metallic Ni favor H adsorption and NiO/Ni(OH)_2_ favor OH_ads_ adsorbed hydroxyl species.

**Figure 4:**
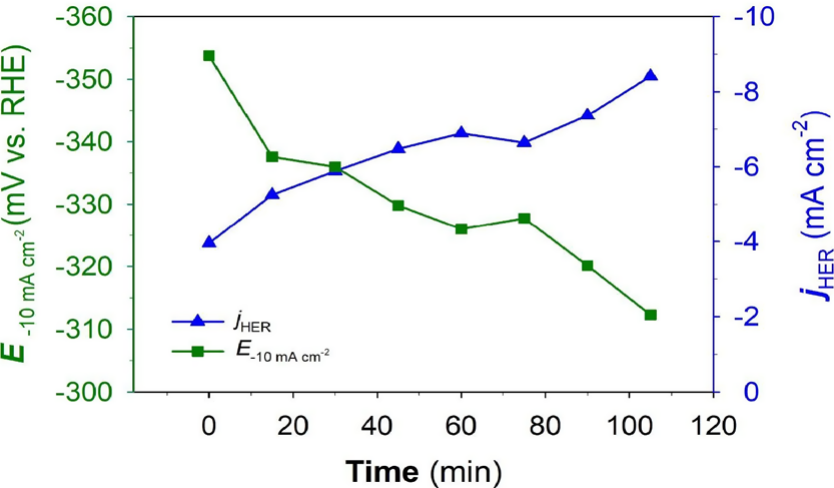
*j*_HER_ and *E*_–10.0mAcm_^–2^ vs ultrasonication durations at the Ni(poly) electrode in N_2_-saturated 1.0 M aqueous KOH solution before and after US at various durations of the ultrasonic treatment.

The overpotential required to achieve a current density of −10.0 mA cm^–2^ (*E*_–10.0mAcm_^–2^) for the Ni(poly) electrode before and after the ultrasonic treatment is shown in Table 3. According to the table, the ultrasonic treatment has significantly lowered the overpotential needed to reach −10 mA cm^–2^ from −353 mV vs RHE before ultrasonication to −312 mV vs RHE after 105 min of ultrasonication; thus, the overpotential is decreased by 41 mV. The plot of *j*_HER_ and *E*_–10.0mAcm_^–2^ vs ultrasonication time is shown in Figure 4. Overall, it is observed that increasing the ultrasonication duration enhances the rate of HER on the Ni(poly) electrode while also decreasing the overpotential at −10.0 mA cm^–2^.

The HER mechanism in an alkaline medium has been discussed above and presented in Table 1. The reaction follows the Volmer–Heyrovsky or the Volmer–Tafel mechanism; in each mechanism, either the first reaction or the second one is the rate-determining step (*rds*). In the case of planar or near-planar electrodes, one can successfully deduce which reaction is the *rds* by analyzing the value of the Tafel slope.^43−45^ According to the existing literature, the Tafel slope of the HER taking place at polycrystalline Ni-based materials in aqueous alkaline electrolyte solutions is ca. 116–120 mV dec^–1^ implying that the *rds* is the Volmer step.^45−49^ However, lower values (e.g., <100 mV dec^–1^) and higher values (e.g., >140 mV dec^–1^ or higher) of Tafel slopes are sometimes reported for Ni materials possessing nonplanar shapes and extended surface areas or significant porosity, such as porous Ni and Raney Ni.^50−54^ In our study, the Tafel plots before and after ultrasonication of the Ni(poly) electrode show one quasi-linear region, and the Tafel slope values vary from 105 mV dec^–1^ before ultrasonication to 120 mV dec^–1^ after 105 min of ultrasonication; these values indicate that the HER follows the Volmer–Heyrovsky mechanism with the Volmer reaction being the *rds*. This is an important observation because it indicates that the ultrasonic treatment does not modify the HER mechanism at the Ni(poly) electrode but increases the rate (current density).

### Pulsed Ultrasound

**Figure 5:**
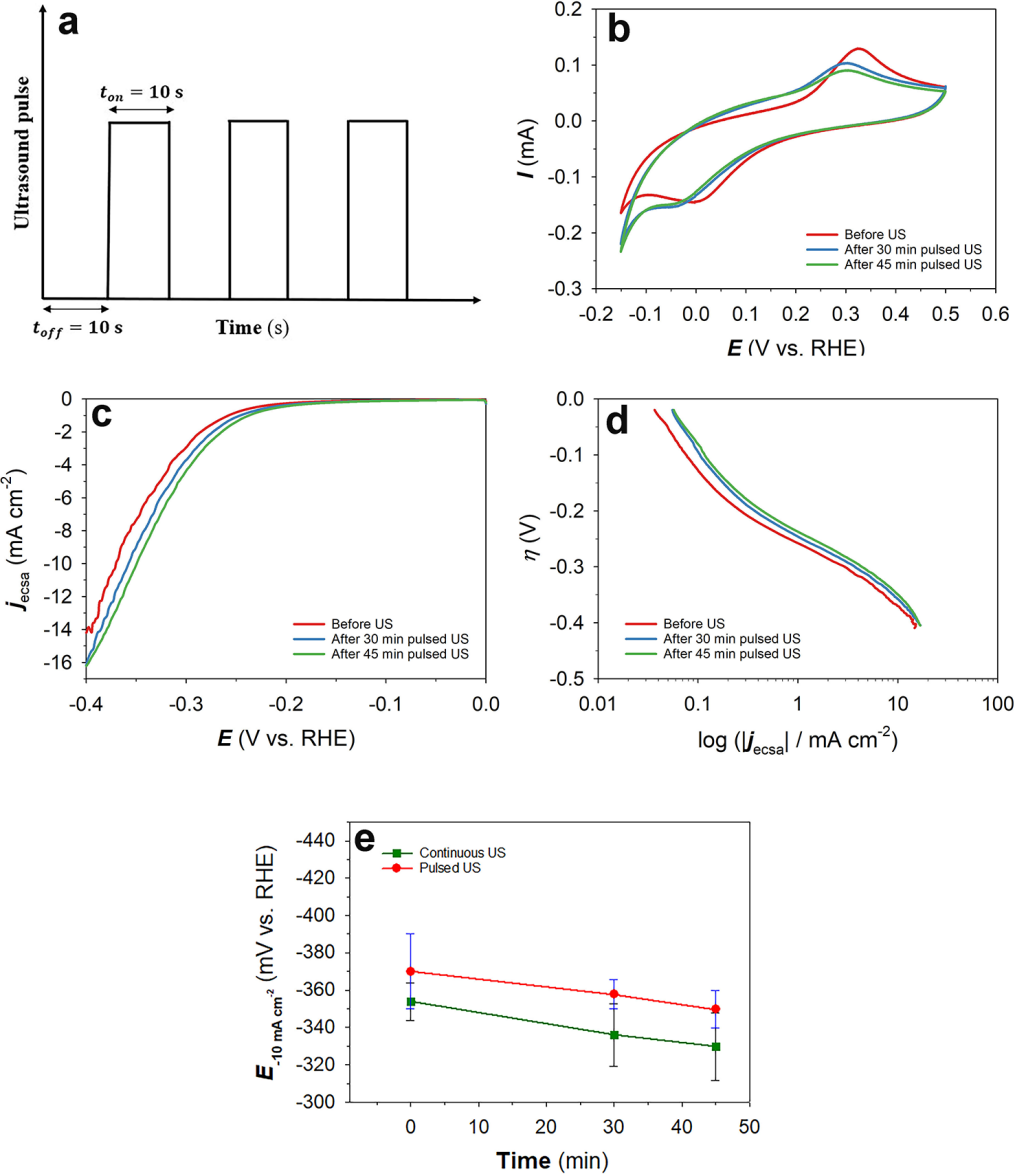
(a) Schematic of ultrasound pulses, (b) CV transients, (c) LSV curves, (d) Tafel plots, and (e) comparison of the overpotentials at −10.0 mA cm^–2^ (*E*_–10.0mAcm_^–2^) for pulsed and continuous modes of the Ni(poly) electrode in N_2_-saturated 1.0 M aqueous KOH solution acquired at *T* = 298 K before and after pulsed ultrasound (US) (*A*_ecsa_ = 0.350 cm^2^).

Continuous use of ultrasonication (20–100 kHz) in sonoelectrochemical processes requires a considerable amount of energy. To overcome this issue and to reduce the energy requirement while still benefiting from the phenomena associated with the use of ultrasonication, different strategies can be employed, such as using pulsed ultrasound.^55−59^ The pulsed ultrasound used in an electrochemical reaction could reduce significantly ultrasonic cavitation erosion at the electrode surfaces, resulting in longer stability of the electrodes.^59^ Here, we performed additional experiments by applying pulsed ultrasound (24 kHz, 60% acoustic amplitude, 44 ± 1.40 W) in order to investigate whether it is possible to activate the Ni(poly) electrode in situ. In our study, the pulsed ultrasonic treatment times were 30 and 45 min in duration, which were equivalent to ca. 15 and 22 min of continuous ultrasonic treatment, as determined using eq 9:
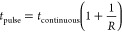
9where *t*_pulse_ is the pulsed ultrasound time, *t*_continuous_ is the continuous ultrasound time, and *R* is the ratio of *t*_on_/*t*_off_ for the pulse wave. The pulsed mode was *t*_on_ = 10 s and *t*_off_ = 10 s. Figure 5a shows a schematic of the on and off ultrasonic pulses as a function of time. Figure 5b presents CV transients of the Ni(poly) electrode before and after the pulsed ultrasonic treatment in the −0.15 ≤ *E*_app_ ≤ +0.50 V vs RHE range acquired at a potential scan rate ν = 100 mV s^–1^ and at *T* = 298 K. It can be observed that the oxidation peak associated with the anodic formation of α-Ni(OH)_2_ after the pulsed ultrasonic treatment decreases and shows the same features as the CV profiles for the continuous ultrasonication, thus in agreement with our results reported above for the continuous ultrasonic treatment. *A*_m_ determined from the charge (*Q*) associated with the formation of α-Ni(OH)_2_ after pulsed ultrasonication shows a slight decrease from 0.35 cm^2^ before the pulsed ultrasound to 0.33 and 0.29 cm^2^ after 30 and 45 min pulsed ultrasonic treatment, respectively. The LSV transients of the Ni(poly) electrode in 1.0 M aqueous KOH solution in the 0.00 ≤ *E*_app_ ≤ −0.60 V vs RHE range at a potential scan rate *ν* = 0.30 mV s^–1^ and at *T* = 298 K before and after 30 and 45 min of the pulsed ultrasound treatment are shown in Figure 5c. It can be observed that the current density of −10.0 mA cm^–2^ is obtained at a potential of ca. −370.0 mV before the pulsed ultrasonic treatment, as compared to −360 mV and – 345 mV after 30 and 45 min of the pulsed ultrasonic treatment, respectively. A decrease of the HER overpotential needed to reach a given current density (here −10.0 mA cm^–2^) could be due to partial oxidation of the Ni(poly) electrode brought about by the pulsed ultrasound. Figure 5d presents Tafel plots obtained using the LSV curves. The Tafel slopes remain almost unchanged before and after the pulsed ultrasonic treatment and are found to be *b* = 106 mV dec^–1^. Figure 5e shows the overpotentials required to achieve a current density of −10.0 mA cm^–2^ (*E*_–10.0mAcm_^–2^) at various ultrasonication durations under the continuous and pulsed US modes. It can be observed that the pulsed ultrasonic treatment does not greatly affect the overpotential values when compared to the continuous ultrasonic treatment (a difference of ∼15 to 20 mV across the ultrasonication durations used), although one may argue that the pulsed US mode could save energy and reduce the eventual cost of electrode activation if this treatment were applied on an industrial scale. In order to prove this statement, a techno-economics assessment needs to be undertaken, which is outside the scope of this study.

## Conclusions

In this investigation, we developed a method to in situ activate Ni(poly) electrode in 1.0 M aqueous KOH electrolytes toward the HER by applying continuous and pulsed ultrasonic (24 kHz, 60% amplitude, 44 W). The electrochemical measurements combined with scanning electron microscopy characterization were used to explain the ultrasonic activation of the Ni(poly) electrode surface.

It was observed that increasing the duration of ultrasonic treatment prior to electrochemical testing improves the HER activity and the longer the ultrasonication treatment, the higher the electrocatalytic activity. We also showed that the HER may occur on both the metallic and oxidized sections of the Ni(poly) electrode but at different rates. Furthermore, the SEM images and the electrochemical results demonstrate that the electrochemical surface area of the Ni(poly) electrode is not greatly affected by ultrasonication and the ultrasonication treatment does not modify the HER mechanism of the Ni(poly) electrode in our conditions. The findings presented in this study demonstrate a novel use of ultrasonication to enhance the electrocatalytic activity of polycrystalline Ni toward the HER and open a new research direction for ultrasonic activation of Ni-based electrodes in AWE.

## Materials and Methods

Electrochemical experiments were performed using a potentiostat/galvanostat (BioLogic-SP 150) in a three-electrode configuration. The CV and LSV experiments were performed using a double-jacketed sonoelectrochemical cell. Ultrasound generates heat, and it is necessary to circulate cooling water to keep the bulk electrolyte temperature (*T*) constant. In this regard, a refrigerated circulator (JULABO, Germany) was connected to the sonoelectrochemical cell to maintain the temperature at 298 ± 1 K. This sonoelectrochemical cell employed in this study, also called the *Besançon cell*, has been described in detail elsewhere.^23,30,31^ The working electrode (WE) was a replaceable, disc-shaped solid electrode (E5TQ series, Pine) which in this case was polycrystalline Ni (99.99% in purity, Goodfellow; Ø = 5.00 mm) having a geometric surface area (*A*_geom_) of 0.196 cm^2^. The reference electrode (RE) was a custom-made reversible hydrogen electrode (RHE).^62^ All potential values in this work are reported with respect to the RHE. A Ni mesh (40 mesh woven from 0.13 mm diameter wire, 99.99% in purity, Alfa Aesar, Germany) was cut out in a rectangle shape (20.67 × 10.76 mm^2^) and used as a counter electrode (CE). Its surface area was at least 10× greater than that of the WE.^60,61^ The distance between the ultrasonic probe and the WE was ca. 30 mm (half the length of the ultrasound wave from the oscillator).^30,31^ The experimental setup is shown in Figure S1. The experiments were carried out in 1.0 M (pH = 13.7) aqueous KOH (Sigma-Aldrich, 99.99% in purity) solution outgassed using ultrahigh-purity N_2_(g) (99.999% in purity). All solutions were prepared using ultrahigh-purity water (Millipore, 18.2 MΩ cm in resistivity). The temperature of the electrolyte was measured with a Fluke 51 digital thermometer fitted to a *K*-type thermocouple. Hydrogen peroxide solution (H_2_O_2_) was prepared by diluting 30% w/w H_2_O_2_ (Sigma-Aldrich) in ultrahigh-purity water (UHP).

The nickel mesh used to construct the CE was degreased in acetone (>99.5%, VWR chemicals) for at least 30 min and then repetitively rinsed using UHP water followed by rinsing with UHP water under ultrasonication conditions. Before each experiment, the disc-shaped Ni(poly) WE was mechanically polished using alumina suspension (down to 0.05 μm, Buehler Micropolish) to obtain an oxide-free and mirror-like surface. Afterward, the WE was rinsed with UHP water, ultrasonicated in UHP water for ca. 30 s, and finally rinsed in UHP water. All the glassware was cleaned employing the standard cleaning procedure described in detail elsewhere.^62^

To investigate the performance of the Ni(poly) electrode toward the HER in the 1.0 M aqueous KOH solution, a series of LSV experiments were performed in the potential region of 0.00 ≤ *E*_app_ ≤ −0.60 V vs RHE at the potential scan rate of ν = 0.30 mV s^–1^ before and after applying ultrasound, respectively.

For all LSV experiments, the potential values were *IR*-corrected using the eq 10:
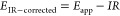
10where *I* is the measured current and *R* is the electrolyte resistance between the WE surface and the RE, measured for each experiment. The *R* value was determined by EIS in the high-frequency region from the value of the *real* impedance (*Z*′) where the *imaginary* impedance (*Z*″) is zero in the Nyquist plot. The EIS experiments were performed in the 100 kHz to 0.1 Hz frequency (*f*) range with a voltage perturbation of +10 mV at an applied potential of *E* = −0.20 V vs RHE at *T* = 298 K.

For all sonoelectrochemical experiments, ultrasound was provided by an ultrasonic probe (Hielscher UP200S, *f* = 24 kHz, 200 W at 60% fixed amplitude, the tip Ø = 14 mm, and the tip area = 153.94 mm^2^ (1.5394 cm^2^)). The calorimetric power (*P*_calorimetric_) was determined calorimetrically using the methods of Margulis et al.^63^ and Contamine et al.^64^ (eq 11) and was found to be 44 ± 1.40 W. The ultrasonic intensity (calorimetric power divided by the surface area of the ultrasonic probe tip) was found to be ca. 28.60 ± 0.90 W/cm^2^.
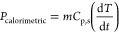
11where *m* is the mass of water (g), *C*_p,s_ is the specific constant pressure heat capacity of water (J g^–1^ K^–1^) and  is the temperature increase after ultrasonication time *t*.

The surface morphology and chemical composition of the Ni(poly) electrode before and after ultrasonication were studied using a scanning electron microscope Zeiss-Ultra 55-FEG-SEM operating at 10 kV accelerating voltage.

## Supporting Information Available

Sonoelectrochemical setup; water sonolysis that includes the major chemical reactions; the effect of ultrasound on the surface roughness of aluminium (Al) foil; and dosimetry measurement of the sonoelectrochemical cell including UV–vis spectra of the peroxotitanium(IV) complex formed by mixing ultrasonicated 1.0 M KOH (24 kHz, *P*_calorimetric_ = 44 ± 1.40 W, 298 K) with TiOSO_4_ solution (PDF)
