# Supplementary material for: In Situ Sonoactivation of Polycrystalline Ni for the Hydrogen Evolution Reaction in Alkaline Media
Source: ACS Appl Energy Mater. 2023 Apr 21;6(9):4520–9. doi: 10.1021/acsaem.2c02443 (PMC10170477; doi:10.1021/acsaem.2c02443)
Supplement: Supplementary file 1 — ae2c02443_si_001.pdf [file ae2c02443_si_001.pdf]

Supporting Information

In-situ Sonoactivation of Polycrystalline Ni for the Hydrogen Evolution Reaction (HER) in Alkaline Media

Faranak Foroughi[1], Marina Tintor[2], Alaa Y. Faid[3], Svein Sunde[3], Gregory Jerkiewicz[1,2*], Christophe Coutanceau[1,4,5,6], Bruno G. Pollet[1,6*]

[1]Hydrogen Energy and Sonochemistry Research Group, Department of Energy and Process Engineering, Faculty of Engineering, Norwegian University of Science and Technology (NTNU), NO-7491 Trondheim, Norway

[2]Department of Chemistry, Queen's University, 90 Bader Lane, Kingston, Ontario, K7L 3N6, Canada

[3]Electrochemistry Research Group, Department of Materials Science and Engineering, Faculty of Natural Sciences. Norwegian University of Science and Technology (NTNU), NO-7491 Trondheim, Norway

[4]Catalysis and Non-Conventional Medium group, IC2MP, UMR CNRS 7285, Université de Poitiers, 4 Rue Michel Brunet, 86073 Poitiers Cedex 9, France

[5]French Research Network on Hydrogen (FRH2), Research federation n°2044 CNRS, France

[6]Green Hydrogen Lab, Institute for Hydrogen Research, Université du Québec à Trois-Rivières, 3351 Boulevard des Forges, Trois-Rivières, Québec G9A 5H7, Canada

[*]Corresponding authors: bruno.pollet@uqtr.ca (B.G.P.) and gregory.jerkiewicz@queensu.ca (G.J.)

1. Sonoelectrochemical set-up

Figure S1 shows the sonoelectrochemical set-up employed in this study. A double-jacketed sonoelectrochemical cell was used and consisted of the cooling jacket and the inner electrochemical cell. The working electrode (WE) was a replaceable, disc-shaped solid electrode which in this case was polycrystalline Ni having a geometric surface area (A_{geom}) of 0.196 cm^2. The reference electrode (RE) was a custom-made reversible hydrogen electrode (RHE) immersed in the same aqueous electrolyte solution as that in the WE compartment, and the counter electrode (CE) was a Ni mesh (the surface area of the Ni mesh was 10 times greater than that of the WE). The three electrodes were immersed in 1.0 M aqueous KOH electrolyte and placed inside the inner electrochemical cell. The ultrasound was transmitted using an ultrasonic probe (horn). The ultrasonic horn was separated from the electrolyte solution in the inner electrochemical cell. The WE was facing the ultrasonic horn, thus was in a so-called "face-on" geometry. The base of the inner electrochemical cell was at a distance, *d* (30 mm) from the ultrasonic horn.

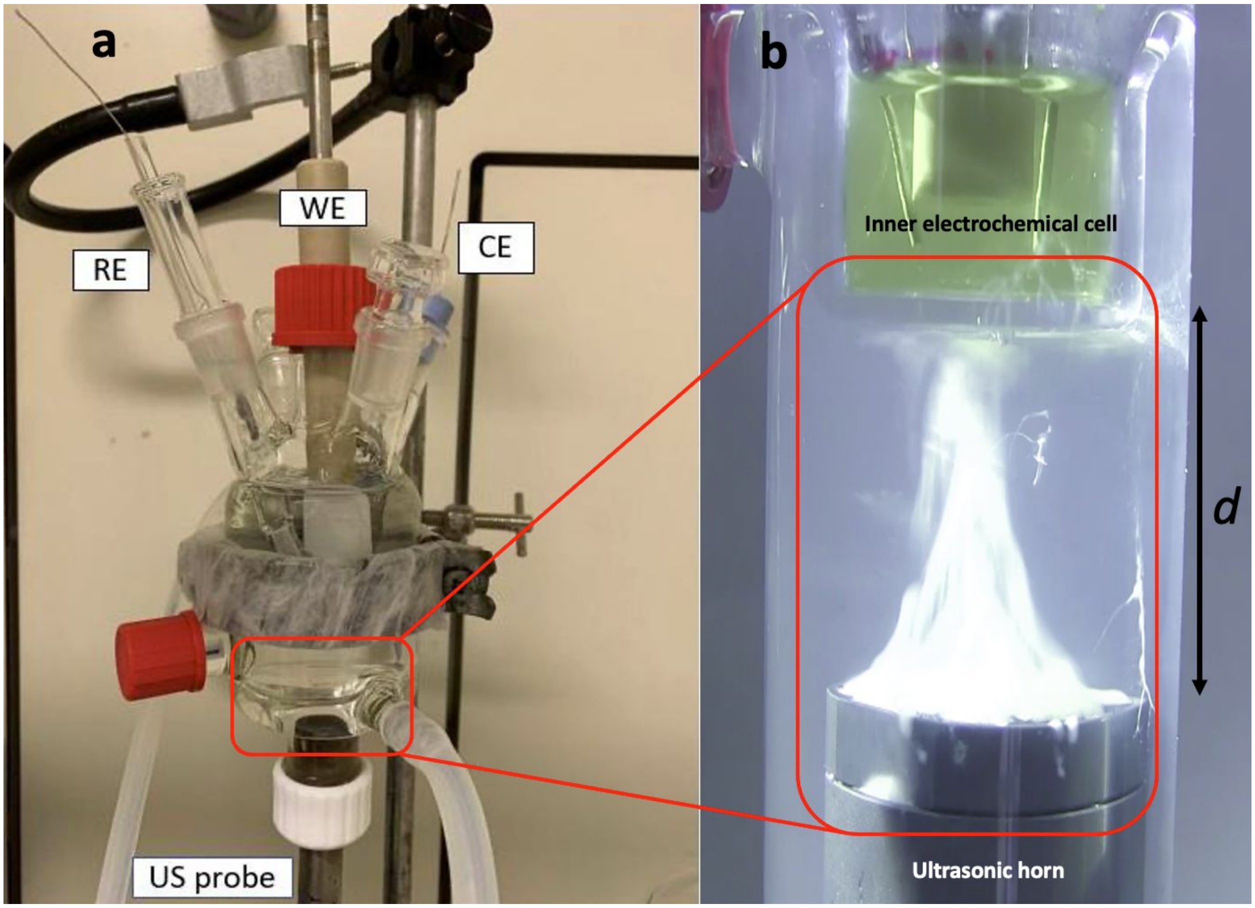

Figure S1. (a) A double-jacketed sonoelectrochemical set-up. WE, CE, and RE refer to the working, counter, and reference electrodes, respectively; (b). The base of the inner electrochemical cell was at a distance, *d* (30 mm), from the ultrasonic horn.

2. Water sonolysis

The collapsing cavitation bubble can generate high temperatures up to 5,000 K and high pressures up to 2,000 atm.[1,2] As the high temperature and pressure generated by the bubble collapse, the solvent vapour and gas molecules generate highly reactive radicals and other species, such as $H^•$ and $OH^•$ radicals, O_3, H_2O_2 and O atoms through endothermic chemical reactions. The hydroxyl radical ($OH^•$) is the most dominant species generated in sonochemical reactions. It is

well known that the $OH^•$ radical and O atoms reacts with H_2O to produce H_2O_2. A few possible reactions are shown in equations (1)-(4).

$$H_2O \rightarrow H^• + OH^• \quad (1)$$

$$H^• + OH^• \rightarrow H_2 + O \quad (2)$$

$$OH^• + OH^• \rightarrow H_2O_2 \quad (3)$$

$$H^• + H^• \rightarrow H_2 \quad (4)$$

3. Effect of ultrasound (US) on the surface roughness of aluminium (Al) foil

It is well-known that ultrasound can cause either macroscopic mechanical effect (damage) due to the intense sound field or microscopic or nanoscopic ones caused by the collapse of cavitation bubbles and the impact of the shock waves. When a metal is ultrasonicated, a cavitational erosion phenomenon is observed leading to surface damages and deformations.[1-3] In this regard, a piece of Al foil was ultrasonicated for 10 min in 1.0 M aqueous KOH in the same sonoelectrochemical set-up as that in which the rest of the experimental work was conducted. Prior to and after ultrasonication, visible light microscopy images of the Al foil were acquired at two different magnifications by microscopy using a WITec alpha 300 R Confocal Raman spectrometer. The images a and b in Figures S2 shows the Al foil before ultrasonication and the images c and d show the Al foil after 10 min of ultrasonication in 1.0 M aqueous KOH solution. A comparison of the four images reveals that the Al foil underwent erosion that gave rise to surface roughening. To understand whether the erosion of the Al foil can be attributed to ultrasonication or to the reaction with the 1.0 M aqueous KOH solution, we also immersed the Al foil for 10 min in 1.0 M aqueous KOH solution without ultrasound (the images e and f in Figure S2). They show that the Al foil

became eroded and roughened in KOH although no ultrasound was applied. Because the extend of erosive degradation is the same in the images c through f, it may be concluded that the erosion and roughening (most likely erosion-corrosion) is caused by the exposure of the Al foil to the aqueous KOH solution and in the case of our experimental setup is not caused by ultrasonication. The same experiment was performed in ultra-high purity (UHP) water to exclude any corrosion of the Al foil caused by the aqueous KOH solution and the duration of the ultrasonic treatment was also 10 min. The images a and b in Figure S3 present the Al foil before and after 10 min of ultrasonication, respectively, and do not reveal any roughening or pitting. Thus, it may be concluded that under the experimental conditions described in this contribution (they also include the cell geometry and the distance between the ultrasound source and the Ni electrode), there is no surface damage that could be attributed to the ultrasonic treatment and if any damage is observed, then it should be assigned to other phenomena. It should be added that a new Al foil sample was used in each experiment and all samples were prepared from the same batch of Al foil. It should be added that the damage of the Al foil caused by the exposure to the 1.0 M aqheous solution is entirely expected because an as-received Al foil is covered with a layer of Al_2O_3, which is known to have an amphoteric nature and reacts with alkaline media, in this case with the aqueous KOH solution and forms $KAl(OH)_4$, potassium aluminate.

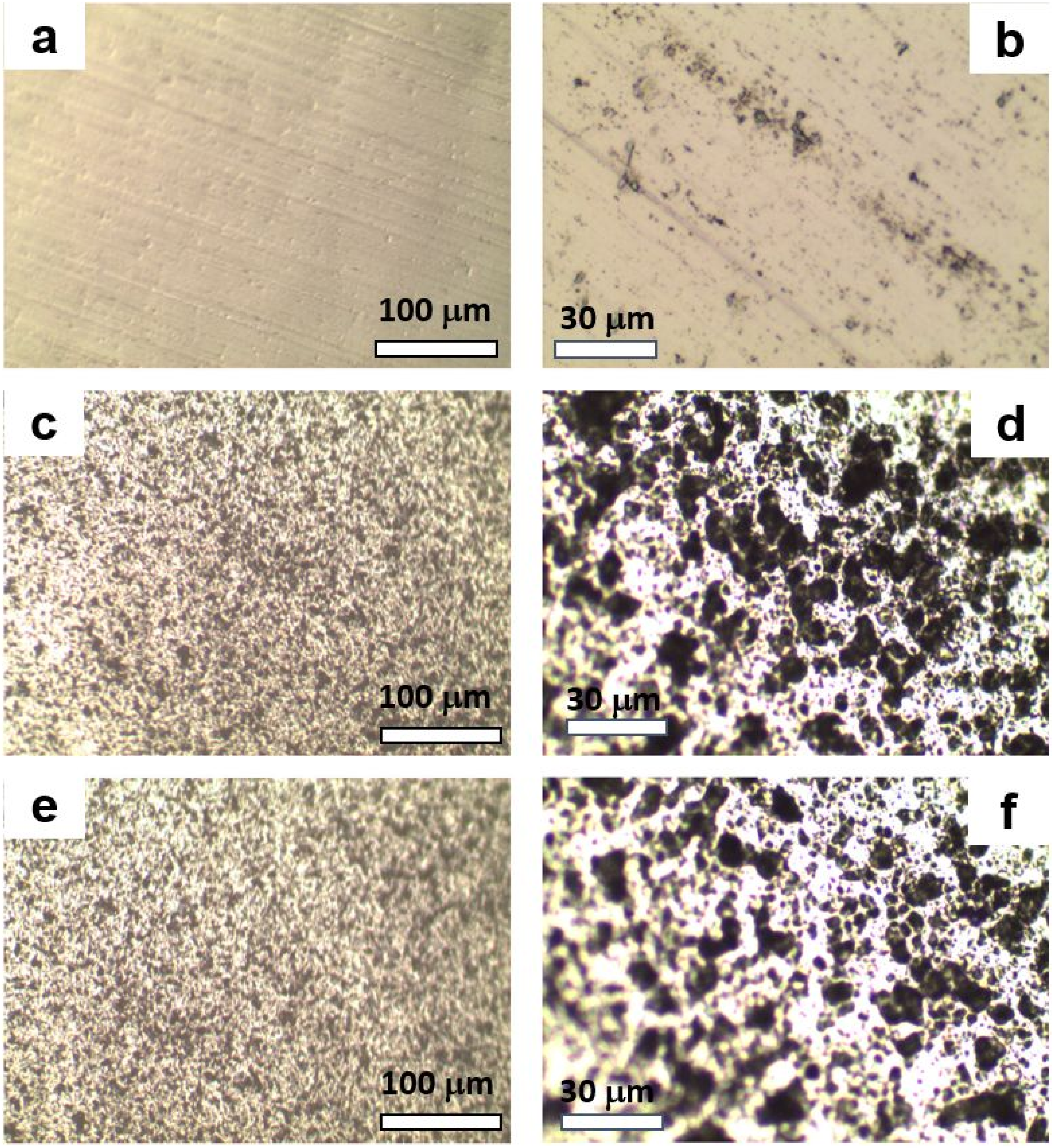

Figure S2. Visible light microscopy images of the Al foil. The images (a) and (b) refer to the Al foil prior to and the images (c) and (d) after 10 min of ultrasonication in 1.0 M aqueous KOH solution. The images (e) and (f) refer to the Al foil after being immersed in 1.0 M aqueous KOH solution for 10 min without ultrasonication.

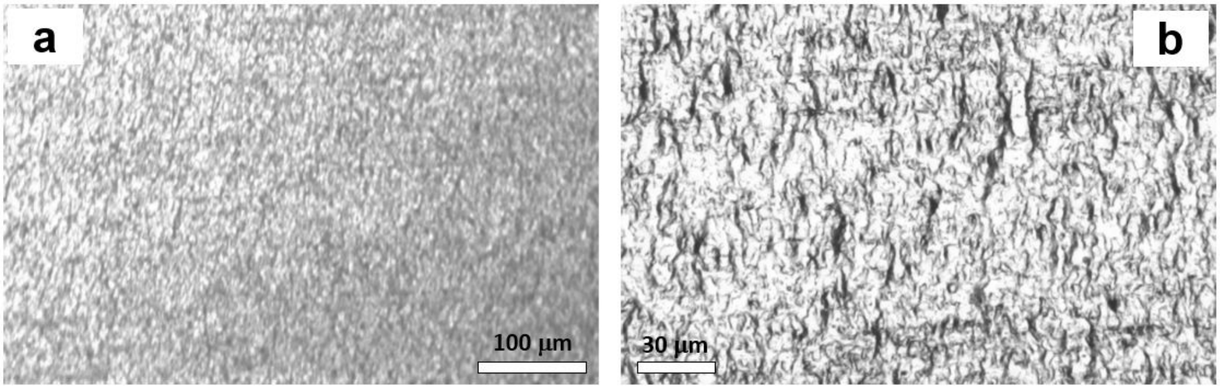

Figure S3. Visible light microscopy images of the Al foil. The images (a) and (b) refer to the Al foil after 10 min ultrasonication in deionized water at two magnifications.

4. Dosimetry experiments

To characterise the sonochemical activity in the sonoelectrochemical cell under the specified experimental conditions (24 kHz, 44 ± 1.40 W, 60% amplitude power, 298 K), dosimetry experiments were performed.[4] In these experiments, 100 mL of 1.00 M aqueous KOH solution were ultrasonicated for 105 minutes at the above-specified conditions. During ultrasonication, a 0.50 mL aliquot of the solution was sampled every 15 min and diluted with a 0.50 mL of $TiOSO_4$ solution (2.00×10^{-2} M, sigma-Aldrich).[5] The *as*-formed peroxotitanium(IV) complex was then analysed using a Thermo Scientific Evolution UV-Visible absorption spectrophotometer (Figure S4). The UV-Visible absorption spectra of the ultrasonicated solution show that peak intensity changes demonstrating the radical formation with ultrasonication time. The radical formation also can be depicted from the rise in absorbance and the ultrasonication time increases.

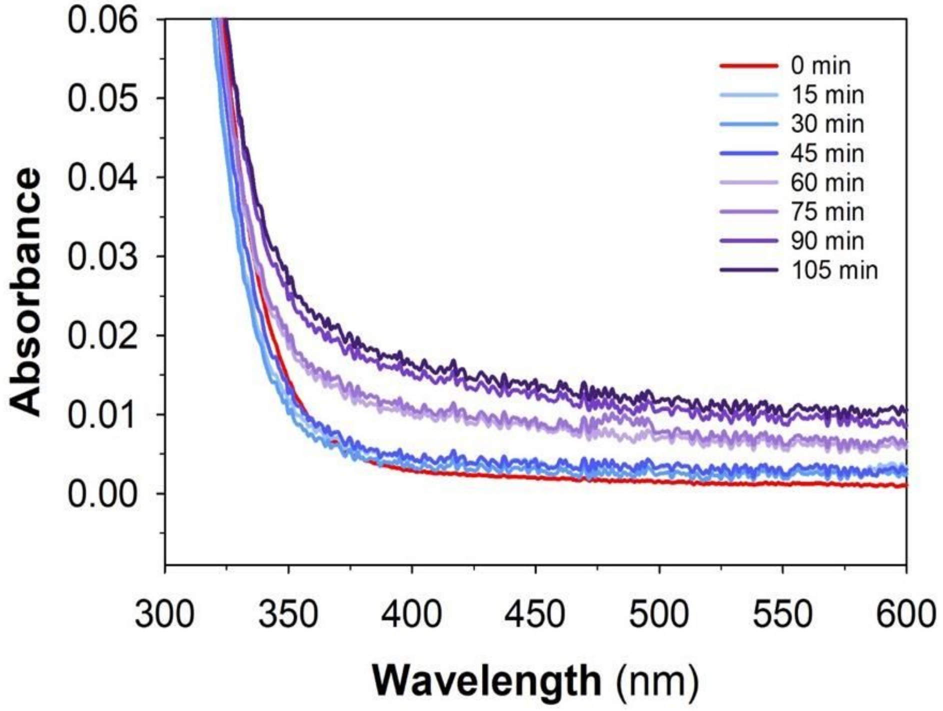

Figure S4. UV-vis absorption spectra of the peroxotitanium (IV) complex formed by mixing sonicated 1.0 M aqueous KOH solution at T = 298 K (24 kHz, P_{US} = 44 ± 1.40 W) with $TiOSO_4$ solution.

References

1. Verdan, S.; Burato, G.; Comet, M.; Reinert, L.; Fuzellier, H., Structural changes of metallic surfaces induced by ultrasound. *Ultrasonics Sonochemistry* **2003,** *10* (4-5), 291-295.
2. Mason, T. J., *Advances in sonochemistry*. Elsevier: 1996. ISBN: 9780080560809
3. Ashokkumar, M.; Grieser, F., Ultrasound assisted chemical processes. *Rev. Chem. Eng.* **1999,** *15* (1), 41-83.
4. Gong, C.; Hart, D. P., Ultrasound induced cavitation and sonochemical yields. *Journal of the Acoustic Society* **1998,** *104* (5), 2675-2682.
5. Dalodière, E.; Virot, M.; Moisy, P.; Nikitenko, S. I., Effect of ultrasonic frequency on H_2O_2 sonochemical formation rate in aqueous nitric acid solutions in the presence of oxygen. *Ultrasonics Sonochemistry* **2016,** *29*, 198-204.
